# Recognising dying: Will artificial intelligence (AI) help improve clinical accuracy?

**DOI:** 10.1016/j.fhj.2026.100516

**Published:** 2026-03-27

**Authors:** Eleni Lester, Simon Tavabie, Nicola White, Ollie Minton

**Affiliations:** a Barts Health NHS Trust, London, UK; b Marie Curie Palliative Care Research Department, University College London Hospitals NHS Foundation Trust, London, UK; c University Hospitals Sussex NHS Foundation Trust, Brighton, UK

**Keywords:** Dying, Palliative care, Prognosis, Uncertainty, Artificial intelligence, Human-AI collaboration

## Abstract

The number of people requiring palliative care in the UK is projected to rise significantly, creating an urgent need for earlier and more systematic recognition of those approaching the end of life. Current clinical markers and tools for predicting prognosis are limited in their accuracy, and prone to human and systemic biases. Artificial intelligence (AI) offers potential to improve prediction of deterioration and dying, and early studies suggest that it may support timely interventions and advance care planning. However, integration of AI must prioritise data integrity, accountability and minimising the amplification of existing inequities. Crucially, recognising dying remains a fundamentally human task with ethical, relational and existential dimensions that AI cannot replicate. Successful implementation will depend on thoughtful human–AI collaboration that strengthens clinical insight without compromising the compassionate, person-centred approach that is central to palliative care.

## By 2030–2035, AI will almost certainly surpass human clinicians in recognising dying

It’s a bold assertion – and ChatGPT™ continues, suggesting that AI has already surpassed human capability in certain clinical contexts. But before we call for the immediate curtailing of palliative care training, leaving existing specialists to explore the UK’s benefits system, it is worth pausing to ask: what do we mean by ‘recognising dying’, why does it matter and how might the role of human clinicians evolve alongside advancing technologies and AI?

## Why recognising dying matters

The need for earlier recognition is vital: too many people are dying in crisis, with an overreliance on urgent and unplanned care, within services that are already overstretched.^1^^,^^2^ For those who die in a hospital setting, their needs often go under-identified and unmet, despite attempts at improving these standards of care.^1^^,^^3^

These challenges are only set to intensify. Owing to a growing, ageing and increasingly multimorbid population, the number of people in the UK requiring palliative care is projected to increase by up to 42% by 2040–2045, with 100,000 more people dying annually.^4^ Employing systematic approaches to enable earlier recognition of adults approaching the end of life is fundamental for optimising patient care, carer support, service provision and resource management, as we navigate these demographic shifts.^5^^,^^6^

## What do we mean by ‘recognising dying’?

To recognise dying, we must first understand what we mean by the word ‘*dying’*. Whereas death is a state – the permanent cessation of life experienced not by the individual person, but rather by the people who knew them (excluding any concepts of the afterlife) – dying is a process that marks the transition between life and death.

Aside from the bio-reductionist view that we are all dying from our mid-20s onwards, the beginning of dying is often taken to coincide with the onset of a terminal, progressive condition. McCartney and Trau describe this condition as being ‘caused by injury, disease, or illness from which, to a reasonable degree of certainty, there can be no restoration of health, and which, absent artificial life-prolonging procedures, will inevitably lead to natural death’.^7^

Such a definition can, however, become inappropriate or ethically obscure in cases where people live with a terminal condition for much of their lifetime. To understand what is meant by dying, therefore, it is vital to recognise dying as a multidimensional process with bio-psycho-socio-spiritual components.^8^ In much the same way that illness is considered the subjective experience of disease, dying can be thought of as the subjective experience of living with a terminal, progressive condition and approaching death, making it unique to each individual’s circumstances.

The dying process can be short, or prolonged over many years. In the UK, those with a terminal, progressive disease who are expected to have a prognosis of less than a year are described as approaching the ‘end of life’.^9^ As a person approaches death, each stage of transition – from living with terminal illness, to approaching end of life, to actively dying – may bring with it changes in the place, level and goals of care.^10^ Recognising when someone may be entering the last months, weeks, days or hours of life can therefore help clinicians support their patients through these transitions and guide personalised end-of-life care.

## Current clinical prediction markers and tools

### Illness trajectories

Three distinct physical trajectory categories have been recognised in palliative care – cancer, organ failure and frailty/dementia.^8^ These trajectories provide useful conceptual frameworks that outline expected patterns of overall physical decline, acute episodes and healthcare interaction.

**Fig. 1:**
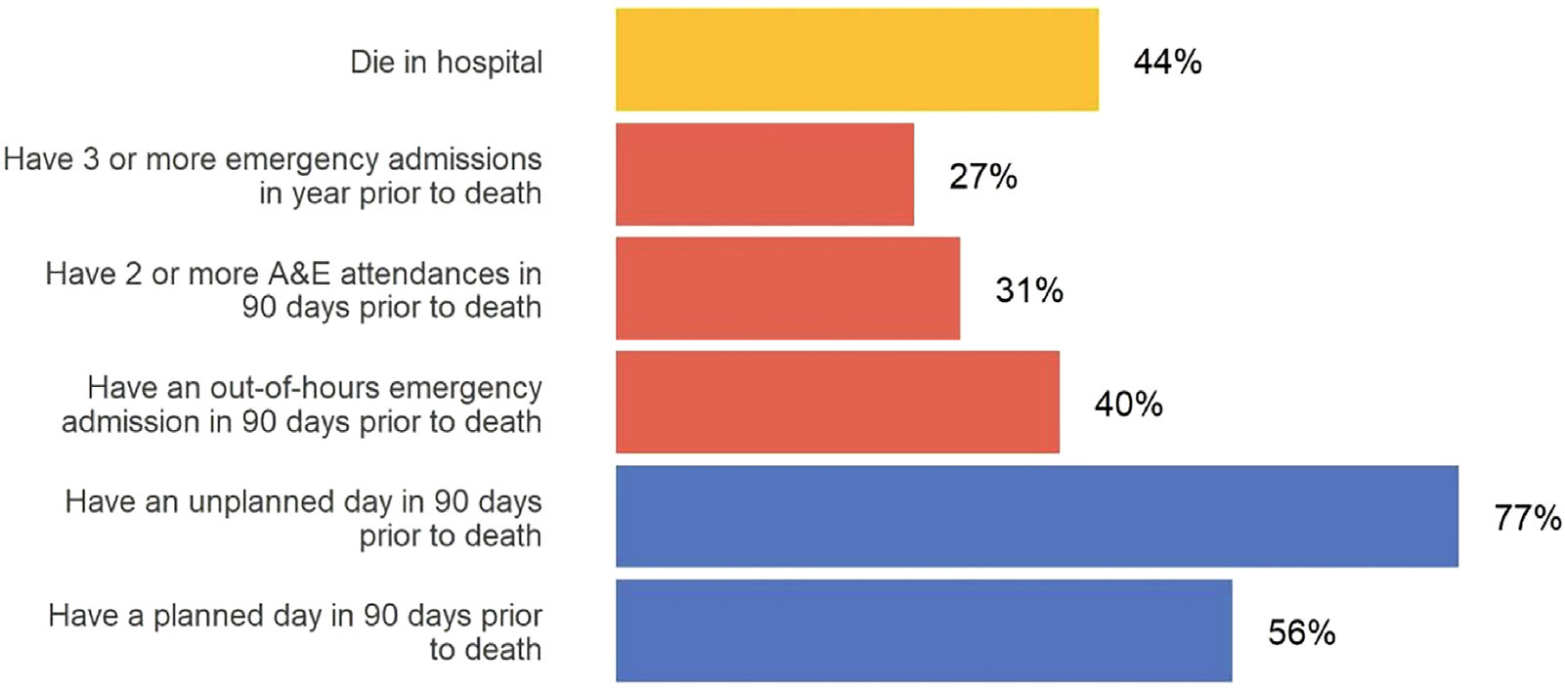
Percentages of decedents who experience each outcome, from *A picture of end-of-life care in England*.^4^

Interactions with healthcare typically increase as a person approaches death. *A picture of end-of-life care in England* showed that 44% of people died in hospital, with 40% of these following at least one out-of-hours emergency admission in the 90 days prior to death.^4^ This builds on earlier research showing that approximately one-third of hospital inpatients are in their last year of life.^11^ Recognition of dying can be particularly challenging in organ failure, where repeated episodes of acute deterioration, hospitalisation and recovery are common, making it difficult to predict which episode will be the last^8^ (Fig. 1).

With the above in mind, it may not be unreasonable to suggest that the question *‘Could they be dying?’* should be considered for any patient requiring an unplanned or emergency hospital admission, particularly in the context of cancer, organ failure or frailty/dementia.

Evidence of psycho-socio-spiritual change, decline or distress might also prompt this question. A prospective longitudinal study of cancer patients used multiple scoring systems to track patient-reported wellbeing across the multiple dimensions of dying. In doing so, they captured trajectories not only of physical decline, but also of psychological, social and existential distress. The authors suggest that such tools can help identify ‘points of accelerated deterioration’, enabling clinicians to predict and anticipate key transitions in the illness and dying process.^12^

In clinical practice, deterioration trajectories and clinical change are also commonly used as a proxy for predicted prognosis. When a patient is recognised as dying and their relative or loved one inevitably asks – *How long left?* – the pattern and rate of clinical deterioration is often used by clinicians to guide prognostication. As an example, deterioration on a day-by-day basis often suggests that someone may be in their last hours to days.

### Assessment tools

Several clinical tools have been specifically designed to aid recognition of dying. The Gold Standards Framework (GSF) Proactive Identification Guidance (PIG) tool begins by prompting the ‘surprise question’: ‘Would you be surprised if this patient died within the next 12 months?’^6^ It then sets out indicators of deterioration, both general and specific to disease type. The GSF also uses a colour-based coding system to classify patients by need and prognosis: red (last days of life), amber (deteriorating/weeks prognosis) and green (unstable/months prognosis). The Supportive and Palliative Care Indicators Tool (SPICT) similarly prompts identification of deteriorating health indicators to support earlier recognition.^13^

Other tools, although not designed specifically with this purpose in mind, have been shown to correlate with prognosis. These include the Australia-modified Karnofsky Performance Scale (AKPS) and Palliative Performance Score (PPS).^14^

The Integrated Palliative Care Outcome Scale (IPOS) is a validated patient-reported tool that maps physical, psychological, social and spiritual needs in advanced illness.^15^ In a recent prospective observational study of hospitalised patients with heart failure, higher palliative care needs (PCN) as determined by elevated IPOS scores were shown to be independently associated with higher risk of mortality, but did not correlate with other markers of heart failure severity such as the New York Heart Association (NYHA) score. This illustrates the potential for use of IPOS in prognostication.^16^

### Clinical signs in active dying

When predicted prognosis is measured in hours to days, patients are described as ‘actively dying’ or in ‘the end stage’.^9^^,^^10^ Specific clinical signs associated with ‘active dying’ include changes in breathing pattern (Cheyne–Stokes breathing), increased agitation, reduced responsiveness, reduced eating/drinking, frequent sleeping, mottled or cold skin, and noisy respiratory secretions.^17^ One study found that in clinicians with objectively stronger prognostic ability – meaning that they were found to be more likely to successfully recognise whether a patient is in the last 72 h of life – Cheyne–Stokes breathing and low PPS score were the most influential factors.^2^

## Why recognising dying is difficult

### Limitations of current clinical prediction tools

• *Suitability:* lllness trajectories as described by Murray *et al* have provided a useful conceptual framework;^8^ however, with increasing clinical complexity and the introduction of more advanced palliative treatments such as immunotherapy, their utility may be diminishing.^18^
• *Accuracy:* The ‘surprise question’ is overly broad and relies heavily on user experience. Its accuracy has been shown to vary widely: a systematic review demonstrated sensitivity ranging from 11.6% to 95.6% and specificity ranging from 13.8% to 98.2% across studies. Among the patients identified as being at risk of dying in the next 12 months, there were about as many ‘false positives’ as ‘true positives’.^19^ In another study, fewer than half of the patients recognised by the SPICT tool as being at risk of deteriorating or dying had died within 12 months.^20^
• *Usability:* A core systemic issue is that none of the tools designed to help clinicians recognise dying are currently integrated into NHS computer systems or available as dedicated applications. The failure to link NHS systems prevents coordinated care, complicates communication after dying is recognised and hinders the ability to collect the necessary data to improve tool efficiency. Consequently, the number of people recorded on palliative care registers varies widely by place: whereas some regions in England have all decedents registered, others have only one in three.^4^

### Human–system factors

Even if we were to have more accurate clinical tools to guide prognostication, the recognition of dying remains heavily reliant on human factors, systems and culture, some examples of which are outlined here:

• *Role definition:* The age-old question of ‘whose role is it?’ remains contested. It often still falls to senior doctors to make decisions about whether someone is dying, despite nurses, healthcare assistants and junior doctors often having closer and more sustained contact with patients.^21^ Significantly different approaches to recognising and communicating dying have also been found between different specialists, for example oncologists versus non-oncologists.^22^
• *Experience, skill and intuition:* Clinical signs of dying may be present, but they will only be recognised if the ‘recogniser’ is sufficiently experienced, confident and supported to act on them. Clinicians who have completed a palliative care rotation describe greater confidence and ability in recognising dying,^21^ underscoring the importance of ongoing education around palliative and end-of-life care principles.
• *Clinical uncertainty and fear:* Clinicians must contend with clinical uncertainty and the fear of getting prognosis wrong. Managing uncertainty in clinical practice is a growing field within research, but can present barriers to decision making in all forms of serious illness.^23^
• *Emotional barriers:* There is often a fear of upsetting the patient or causing them to lose hope, compounded by the fact that patients often want their healthcare provider to be optimistic.^22^ There is also the emotional toll that the clinical relationship has on the clinician; although in many ways knowing a patient for longer might be thought to improve recognition of dying, one observational study found an association between prolonged disease course and a higher rate of unexpected death.^24^
• *Physical and resource factors:* In acute and emergency settings, we are increasingly witnessing how lack of physical space and ‘corridor care’ can be additional barriers to recognising and communicating dying.
• *Socio-cultural factors:* Societal, cultural and religious factors influence whether patients are ready or willing to engage in these discussions. In addition, clinicians are influenced by the cultural factors present within different healthcare settings, specialties, staff groups and levels of acuity.^21^ Language is also implicated; as Colquhoun-Flannery *et al* acknowledge: ‘A culture where dying is not openly acknowledged or even named explicitly contributes to late recognition of dying’.

## Where AI might help and guiding principles for integration

By facilitating more standardised collection of patient-reported data, as well as enhanced pattern recognition of physiological markers and trajectories across the multiple dimensions of dying, AI tools may foreseeably improve the efficiency with which we can predict dying.

Systematic implementation of more advanced technologies, including AI, could in turn drive more effective digital software, such as linked NHS systems, AI triggers and prompts for clinicians, as well as digital tools for patients and carers to be able to feedback and flag concerns. Imagine a future where instead of a *‘Could it be sepsis?’* prompt appearing on screen as you enter a patient record, there is instead a prompt asking: *‘Could they be dying?’* (Fig. 2).

**Figure:**
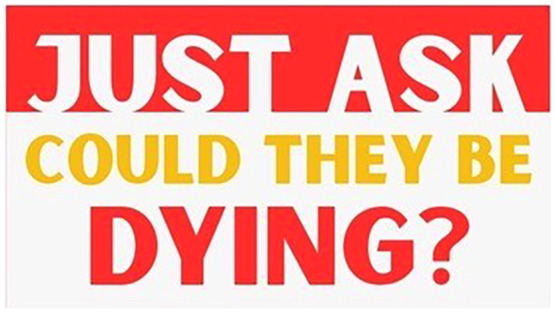
Prompt to consider if someome is sick enough to die.

One such example of AI application in palliative care is the *Palliative Connect* study, which evaluated a mortality prediction model, based on the electronic health record, to trigger palliative care consultation among hospitalised medical inpatients. The intervention was associated with a 74% increase in palliative care consultations, earlier consultation timing, a 38% rise in advance care planning documentation and a 61% increase in community palliative care referrals. However, only 57% of triggered consults were accepted by the primary team.^25^

Despite its potential benefits, implementation of AI in clinical practice is not without its practical and ethical challenges. For human–AI collaboration to be successful, several principles must be rigorously upheld.

### Data integrity

It is vital that AI is trained using reliable data. Currently, the lack of data on specific end-of-life outcomes means that proxy outcomes are used for ‘a bad death’, such as deviation from previously stated preferred place of care (PPC) or death (PPD). There is a need to verify the accuracy of clinical tools in guiding prognostication and to implement their use more systematically; however, this will require significant investment in data collection and research, and risks being onerous not only to clinicians, but also to patients.

It is also important to note how, despite being more easily measurable, quantitative data overly simplify the complex values of, and systems surrounding, the dying person.^26^ An over-reliance on the objectification of subjective, lived experience would sit at odds with the general ethos of palliative care, in which clinicians often do well simply to sit, listen and bear witness.

### Minimising bias

As it stands, worse outcomes, including higher A&E attendances, hospital admissions and dying in hospital, are observed in groups who are younger, male, from Asian or Black ethnicity groups, experiencing deprivation and living alone.^27^ Prognosis prediction influences triage and limits or enables access to services, raising questions of equity, bias and potential racism.^28^ There is a serious concern that existing system and human bias will be absorbed and amplified by AI systems.

### Accountability and trust

Many AI systems operate as ‘black boxes’, generating outputs that are difficult for clinicians and patients to interpret or challenge.^29^ In palliative care, where decisions are ethically complex and closely tied to personal values, opaque decision making risks undermining trust and exacerbating stigma. This is particularly salient in the context of an evolving moral and legal landscape, including debates around assisted dying.

### Human training

By striving for improved prediction of dying, we risk further eroding comfort with uncertainty, a central feature of palliative care. As Colquhoun-Flannery *et al* argue, tools that acknowledge the *possibility* of dying and tolerate inevitable uncertainty may be more helpful than those seeking absolute prognostic precision.^21^ Training should therefore support critical engagement with AI while reinforcing the relational and interpretive skills fundamental to recognising dying (Table 1).

**Table 1:** A hermeneutic prompt set to consider: *‘Could my patient be dying?’*.

Domain	Input	Output (recognition of dying in practice)	Potential role of AI
Longitudinal clinical data, illness trajectory and healthcare utilisation	Repeated unplanned hospital admissions or same-day emergency care; escalating symptom burden; deteriorating clinical markers	Communication of uncertainty and recognition of possibility of dying	Detection of patterns and trends over time; clinical flagging and prompts
Multidisciplinary clinical judgement and senior oversight	Senior MDT review; NEWS and physiological trends interpreted in context	Clear documentation of ceilings of intervention and acknowledged risk of dying	Prompting MDT or senior review
Response to treatment within agreed ceiling of intervention	Clinical deterioration *despite* appropriate treatment at the documented ceiling of intervention	Recognition of dying; parallel planning; consider stopping active treatment and starting Compassionate Care Plan	Flagging treatment non-response
Psychological, social and spiritual concerns	Increasing psycho-socio-spiritual distress in the context of a terminal condition	Recognition of dying as a holistic process; provision of supportive, MDT care; palliative care referral	Surfacing patient-reported outcomes or narrative cues
Patient values, preferences and prior expressed wishes	Advance care plans; previous discussions; known values	Care aligned with patient wishes and proportionality of intervention	Retrieval of documented preferences; streamlined documentation across systems
Clinical uncertainty and need for specialist support	Team disagreement; uncertainty about reversibility; family distress	Palliative care referral; anticipatory prescribing and holistic support	Minimal: trigger prompts only

## What AI cannot replace

Even if AI may soon surpass humans in *predicting* that someone will die, the act of *recognising* dying will remain the domain of the human clinician.

The field of narrative medicine proposes that the meaning of stories is co-constructed: the listener/clinician actively shapes the interpretation of the teller/patient’s story and neither part can therefore be viewed in isolation.^30^ In much the same way, recognising dying in healthcare depends on the active participation of the ‘recogniser’. The clinician must interpret and respond to the patient’s lived experience, but this in turn relies on the experience of the clinician: their intuitions, personality, previous experiences with dying, and the team and environment in which they are working.

Thus, recognising dying has ethical, relational and existential dimensions that transcend the capabilities of AI. Recognising and responding to a dying patient involves shifting clinical focus from ‘how do we treat’ to ‘how do we care’, encompassing honesty, responsibility and accountability. Ethically, we must also ensure equity in the identification and provision of care. The relational dimension centres on communication, close listening and meaning-making, helping patients and families navigate often-distressing emotional and psychological terrain. Although AI can mimic compassion, humans will continue to yearn for human connection in these moments.

Finally, the existential component of dying: human clinicians share mortality with the dying, a fundamental experience that AI cannot replicate. This capacity enables clinicians to witness and make sense of the psychological, social and spiritual dimensions of dying, including concepts of religion, spirituality and the potential for closure or ‘denouement’ of a person’s story.

## Conclusion

Earlier recognition of dying is vital as our healthcare system, already under significant strain, undergoes further population and demographic shifts. Advances in technology and AI may help clinicians predict dying more efficiently and trigger timely prompts and interventions, but any attempts to improve the recognition of dying using AI must be carefully balanced against the risk of amplifying existing biases and inequities, as well as damaging trust in the clinician–patient relationship.

Recognising dying is, and will remain, a profoundly human act. AI cannot interpret the unique, subjective experience of each person approaching death, nor navigate the ethical, relational and existential dimensions that this entails. As technology develops, our challenge will be to integrate AI in ways that support clinicians, while ensuring that the focus remains on caring for the person recognised to be dying, not just predicting their death.

## CRediT authorship contribution statement

**Eleni Lester:** Writing – review & editing, Writing – original draft, Conceptualization. **Simon Tavabie:** Writing – review & editing, Writing – original draft, Visualization, Supervision, Conceptualization. **Nicola White:** Writing – review & editing, Supervision. **Ollie Minton:** Writing – review & editing, Supervision.

## Declaration of competing interest

The authors declare that they have no known competing financial interests or personal relationships that could have appeared to influence the work reported in this paper.
